# Complete genome sequence of the first *Halopseudomonas aestusnigri* strain isolated using an ethylene-α-olefin co-oligomer

**DOI:** 10.1128/mra.00589-25

**Published:** 2025-08-11

**Authors:** Ryo Iizuka, Sotaro Uemura

**Affiliations:** 1 Department of Biological Sciences, Graduate School of Science, The University of Tokyo 13143, Tokyo, Japan; 2 Core Research for Evolutional Science and Technology (CREST), Japan Science and Technology Agency, Toyko, Japan; Montana State University, Bozeman, Montana, USA

**Keywords:** polyolefin, bioplastic, *Halopseudomonas aestusnigri*

## Abstract

We present the complete genome sequence of a *Halopseudomonas aestusnigri* strain isolated from surface seawater using an ethylene-α-olefin co-oligomer. The 3.91-Mb genome includes genes for extracellular and intracellular enzymes that degrade polyolefins and synthesize polyhydroxyalkanoates, suggesting potential for converting polyolefins into biodegradable plastics.

## ANNOUNCEMENT

The bacterium *Halopseudomonas aestusnigri* (1, 2), often isolated from hydrocarbon-contaminated environments, has demonstrated alkane degradation capabilities (3–7), suggesting potential for degrading structurally similar polyolefins. However, no direct reports exist on polyolefin degradation by this species. We report the isolation and genome characterization of a novel *H. aestusnigri* strain from surface seawater that exhibits potential for polyolefin degradation and polyhydroxyalkanoate synthesis abilities.

Surface seawater was collected at Tsukuihama Beach, Kanagawa, Japan (35°11'44.1"N 139°40'02.6"E) on 4 May 2024, and inoculated in artificial seawater containing 0.1% NH_4_Cl (8) and 1% LUCANT HC-40 (Mitsui Chemicals), a lower-molecular-weight polyolefin consisting of ethylene-α-olefin co-oligomers. Following 20 days of incubation at 25°C, a portion of LUCANT substrate was directly streaked onto an agar plate containing artificial seawater medium (8–10). Multiple colonies with different morphologies were obtained, and strain T1L2 was selected based on 16S rRNA gene identification as *H. aestusnigri* (8, 10). The strain was cultured aerobically in artificial seawater medium for 24 h at 25°C for genomic sequencing. Genomic DNA was extracted using Genomic-tip 20/G (Qiagen) and sheared into 10–25 kbp fragments using Megaruptor 3 (Diagenode). The SMRT library was prepared using SMRTbell Prep Kit 3.0 and SMRTbell gDNA Amplification Kit and treated with the Revio Polymerase Kit (all from PacBio). Sequencing was performed using the Revio system (PacBio). SMRT Link (v.13.1.0.221970) (PacBio) was used to trim adapter sequences from the sequencing reads. Circular consensus sequences with average base quality values below 20 were removed. The resulting reads were filtered using lima (v.2.12.0) (https://github.com/pacificbiosciences/barcoding) and pbmarkdup (v.1.0.3) (https://github.com/PacificBiosciences/pbmarkdup) to remove the ultra-low PCR adapters and PCR duplicates, respectively. Filtlong (v.0.2.1) (https://github.com/rrwick/Filtlong) was used to exclude short HiFi reads (<1,000 bp). The remaining reads were assembled to produce a circular contig using hifiasm (v.0.19.8-r603) (11). Taxonomic classification and annotation were performed using DFAST v.1.6.0 (12–14). Genome completeness and contamination were evaluated using CheckM2 (v.1.0.1) (15). Default settings were used for all software.

The T1L2 genome consists of a circular chromosome (Table 1). The genome was closely related to that of *H. aestusnigri* VGXO14^T^ (GCF_002197985.1) (3), with an average nucleotide identity of 97.16%, implying that T1L2 represents a novel strain of *H. aestusnigri*. Automated annotation revealed that the genome encodes a copper resistance system multicopper oxidase with a signal peptide (HspT1L2_29760). A similar oxidase (WP_000018555.1) has been shown to facilitate polyethylene degradation through oxidative processes (16), suggesting potential for extracellular polyolefin fragmentation. The genome also encodes enzymes involved in the aerobic alkane oxidation pathway, including alkane 1-monooxygenases (HspT1L2_13830 and HspT1L2_32840) and downstream oxidation enzymes such as alcohol dehydrogenase (HspT1L2_16440) and aldehyde dehydrogenase (HspT1L2_11870). We identified putative genes for long-chain alkane monooxygenase (AlmA; HspT1L2_09770 and HspT1L2_10380) and Baeyer–Villiger monooxygenase (HspT1L2_31680), likely involved in the oxidation pathway (17). Furthermore, the genome encodes class II polyhydroxyalkanoate synthases (HspT1L2_24620 and HspT1L2_24640). The presence of these enzymes suggests potential for converting polyolefin degradation products into polyhydroxyalkanoates (18, 19). The gene repertoire is conserved across *H. aestusnigri* strains (3, 4, 7, 20), suggesting species-wide potential for plastic-to-bioplastic conversion.

**TABLE 1:** Sequencing statistics and genomic features of strain T1L2

	T1L2
Sequencing results	
Number of reads	31,723
Sum of length (bp)	248,492,333
Read N50 (bp)	9,258
Assembly results	
Number of contigs	1 (Circular)
Total length (bp)	3,908,131
Coverage (×)	63.6
GC content (%)	60.7
Number of protein-coding sequences	3,623
Number of rRNAs	12
Number of tRNAs	64
Coding density (%)	90.1
Completeness (%)	100
Contamination (%)	1.11

## Data Availability

The genome sequence of strain T1L2 has been deposited in GenBank under accession number AP040865.2. Raw sequencing reads are available in SRA under accession number DRR658982. These are associated with BioProject PRJDB20541 and BioSample SAMD00898857.
